# Higher abundance of micro- and nanoplastics in inflammatory tissues from patients with intestinal erosion

**DOI:** 10.3389/fmed.2026.1732146

**Published:** 2026-07-10

**Authors:** Li-Xia Du, Xuan Song, Tian-Chao Deng, Xiao-Qian Feng, Li-Juan Qiao, Hui Xu, Lian-Lian Liu, Yan Xu, Ying Zeng, Hua Qing, Qin Li

**Affiliations:** 1 Department of Gastroenterology, Chengdu BOE Hospital, Chengdu, China; 2 Center of Reproductive Medicine, Chengdu BOE Hospital, Chengdu, China; 3 Department of Pathology, Chengdu BOE Hospital, Chengdu, China; 4 Department of Comprehensive Pediatric Internal Medicine, Chongqing University Three Gorges Hospital, Chongqing, China; 5 Center of Endoscopy, Chengdu BOE Hospital, Chengdu, China

**Keywords:** health risk, inflammatory gut, micro- and nanoplastics, Py-GC/MS, source of exposure

## Abstract

The detrimental effects of micro- and nanoplastics (MNPs) on human health have been the subject of intense research in recent years, but there is little data on how MNPs affect intestinal health. Six participants with colonic or terminal ileal erosion were enrolled, and one erosive tissue sample plus one adjacent non-erosive tissue sample were collected from each participant. Pyrolysis gas chromatography/mass spectrometry (Py-GC/MS) was utilized to determine the MNP content of these tissues after pathological examination investigation verified the existence of inflammation in the erosive tissue samples. Our results revealed that MNPs were present in all intestinal samples, with a total MNP concentration of 1758.15 (664.07) mg/kg in inflammatory tissues and 611.49 (510.64) mg/kg in non-inflammatory intestinal tissues; the difference was statistically significant (*p* < 0.05). Three types of MNPs were identified: polyethylene (PE), polyvinyl chloride (PVC), and polystyrene (PS). PE and PVC exhibited a 100% detection rate in all tissue samples, whereas PS was found in 16.67% of samples. Correlation analysis revealed marginal associations of intestinal MNP levels with age, drinking water source and diarrhea. This work provides preliminary baseline data and hints at a weak link between MNP exposure and intestinal inflammation.

## 1 Introduction

Micro- and nanoplastics (MNPs) are a general term for microplastics (less than 5 mm in diameter) and nanoplastics (1–100 nm in diameter) (1, 2). MNPs that have been discovered in beverages, salt, terrestrial and aquatic creatures, and other dietary matrices imply that humans may be exposed to MNPs via the food chain (3). MNPs can accumulate in intestinal tissues in addition to being absorbed through the digestive system, according to recent studies that found MNPs in human feces and intestinal tissues (4, 5). Besides the digestive tract, MNPs can enter the human body through the respiratory system and pharmaceutical injections. (6) Human placenta, sperm, cardiac tissue, blood, and kidneys have all been found to contain MNPs (7). This suggests that MNPs can build up in various human systems, such as the circulatory, urinary, and reproductive systems. Additionally, MNPs can readily pass through human biological barriers (such as the intestine carrier (8) and the blood-brain barrier (9)) and enter cells, which can have a negative impact on human health (8).

*In vivo* studies on mammals and lower aquatic organisms have shown that MNPs can build up in animal tissues and cause serious organ damage. The detrimental effects of MNP exposure on the gastrointestinal tracts of experimental animals, such as intestinal barrier impairment, inflammatory reactions, and gut microbiota dysbiosis (10), have also been verified by numerous studies. *In vitro* studies on colon cells and colon organoids confirmed that MNPs caused oxidative stress and altered cell metabolism (11, 12). However, little research has been done on the connection between MNPs and intestinal disorders in humans. According to epidemiological studies, human MNP exposure may be linked to dietary practices and cause gut microbiota dysbiosis (13). Higher MNP concentrations found in human colorectal cancer tissues suggest that MNPs may have a negative effect on human health (4). Furthermore, compared to healthy people, patients with inflammatory bowel disease (IBD) have higher fecal MNP levels (5), suggesting a potential association between MNP exposure and intestinal inflammation. Although one of the main routes of MNP exposure is the digestive tract, it is still unclear how MNPs contribute to the pathophysiology of intestinal disorders in humans.

The following were the goals of this initial exploratory study: (1) To measure MNPs in intestinal tissues for the first time using the pyrolysis gas chromatography/mass spectrometry (Py-GC/MS) method; (2) to investigate any possible correlation between MNPs and intestinal inflammation; (3) to look into the lifestyle choices, underlying illnesses, and clinical symptoms that could affect the MNP content in intestinal tissues. Our findings may provide a preliminary foundation for future large-scale validation studies and offer additional evidence for evaluating the potential health impacts of MNP exposure in humans.

## 2 Materials and methods

### 2.1 Collection of gut samples

The ethics committee of Chengdu BOE Hospital approved this investigation on March 27, 2023 (approval no.: 2023002), and all procedures involving human subjects were carried out in compliance with the Declaration of Helsinki’s ethical guidelines. Inclusion criteria: Adult residents of Chengdu who underwent lower gastrointestinal endoscopy at Chengdu BOE Hospital between April and October 2023 and were discovered to have colonic or terminal ileal erosion. Exclusion criteria: ➀ Severe illnesses, like malignant tumors, organ transplant history, human immunodeficiency virus infection, and mental disorders; ➁ Alcohol abuse (defined as a score of more than ten on the Alcohol Use Disorder Identification Test); ➂ Employment in high-risk locations for MNP exposure, such as chemical plants and plastics manufacturing; ➃ Concurrent involvement in additional clinical trials.

Every participant signed an informed consent form, and all human subjects’ right to privacy was protected. Six participants were recruited for this study. All participants had a lower gastrointestinal endoscopy in a clean endoscopic setting. To minimize the influence of external confounding factors on the experimental results, we performed paired comparisons between inflamed and non-inflamed tissues obtained from the same volunteer. Two samples were clamped using disposable metal biopsy forceps: one from the intestinal inflammatory lesion and the other from the normal tissue adjacent to the inflammation in the same participant.

All samples underwent pathological examination and Py-GC/MS analysis for MNP quantification, with sample masses ranging from 0.0015 to 0.0044 g. A blank control group was established for each sample collection procedure to eliminate potential contamination. Samples were preserved in a 10% formaldehyde solution before being transferred to the Department of Pathological Medicine for histopathological examination. In order to prevent the biopsy forceps from coming into contact with the inner wall of the glass vials, samples for Py-GC/MS detection were put straight into vials. All tissue samples and blank controls were quickly sealed in a freezer (−80 °C) before being sent to the lab for MNP analysis.

### 2.2 Sample treatments for histopathological analysis

Tissue samples were embedded in paraffin after being cleared with xylene and dehydrated with ethanol. A semi-automatic rotary microtome (Leica Biosystems, Germany) was used to prepare intestinal tissue sections that were 3 μm thick slices. Hematoxylin and eosin (H&E) were used to stain each section. To obtain stained images, all H&E sections were photographed using a microscope camera (Olympus, Japan).

### 2.3 Sample treatments for MNP analysis using Py-GC/MS

Different solvents can dissolve MNPs of various materials. The extraction method was used to treat the samples (14). Intestinal tissue samples were rinsed with ultrapure water and weighed in Beaker A. Next, 30 g of anhydrous ethanol was added to remove any tiny organic molecules on the samples’ surfaces after the samples were dried. The tissues were then dried again before being pulverized into a fine powder using an agate mortar (Nantong Wanhengxu Chemical, 90 mm). Following dry weight determination, solvent extraction was performed in sequential steps: (1) 10 g of chloroform was added, and after 10 min of ultrasonic treatment, the extracted supernatant was transferred into Beaker B; (2) 10 g of hexafluoroisopropanol was added to the residual sample, followed by ultrasonication for 10 min, and the resulting supernatant was transferred to Beaker B; (3) 10 g of xylene was added to the remaining sample, followed by extraction on a heating plate at 150°C for 10 min, and the extracted supernatant was then poured into Beaker B. All the above steps were repeated three times. Following the completion of the extraction, the supernatant collected in Beaker B was concentrated on a heating plate at 50°C until it was approximately 1 g. The concentrated solution was then transferred into a crucible, dried to constant weight, and prepared for subsequent analysis.

### 2.4 Py-GC/MS analysis of MNPs

This study employs the Zhao et al. (15) method for detection, and the possible plastic particles in the sample were examined using pyrolysis (Py-3030D, Frontier) in combination with mass spectrometry (MS-QP 2020, Shimadzu) and gas chromatography (GC 2020, Shimadzu) (Py-GC/MS). A chromatographic column with the following specifications was used: 30 m of column length, 0.25 mm of inner diameter, and 0.25 μm of film thickness. To achieve chromatographic separation, set the temperature program as follows: 40 °C for 2 min, then ramped up to 320 °C at a rate of 20 °C/min, and maintained at this temperature for another 14 min, for a total program duration of 30 min. The carrier gas was helium with a column flow rate of 1 mL/min. To determine and measure the polymer of the target particles, the ion monitoring (SIM) technique was chosen. The calibration curves for Py-GC/MS polymer quantification are provided in Supplementary Table 1.

### 2.5 Quality assurance and quality control (QA/QC)

The “no plastic” principle was adhered to throughout the entire experiment, including sample collection, storage, processing, and analysis (15, 16). Biopsy forceps were disposable, sterile metal medical instruments, and all experimental consumables were glassware, which was ultrasonically cleaned with ethanol and flame-sterilized prior to use. All reagents were filtered three times through a 0.45 μm PTFE membrane to eliminate background pollution. The sample collection room met the operating room’s cleanliness. During container and reagent preparation, sample processing, and analysis, the experimenters wore cotton lab coats, cotton hoods, and nitrile gloves, with all procedures performed in a fume hood. Prior to sample testing, we separately validated the instrument, reagents and experimental procedures, and no MNPs were detected in any of these controls. This confirms that no plastic contamination was introduced from the instrument, reagents, or experimental operation process.

Blank controls were established to eliminate process-related contamination. Glass bottles were placed in the sampling environment for 30 s to simulate the sample collection process. The blank control containers were filled with a 5 mL aliquot of filtered ultrapure water and then incubated for 24 h straight on a magnetic stirrer. During this time, the PTFE screw caps were opened and closed fifteen times. After gathering blank control solutions, these solutions were combined to create a composite blank solution. Both the experimental samples and the blank controls underwent the same sample processing procedures before Py-GC/MS analysis (see section “2.4 Py-GC/MS analysis of MNPs” for detailed procedures). No MNPs were detected in the blank mixture under the quantitative conditions established for the standards, including limits of detection (LOD) and limits of quantification (LOQ). Accordingly, the measured values reported herein are considered to represent MNPs present in the actual samples.

### 2.6 Statistical analyses

All statistical analyses were performed using IBM SPSS Statistics 27 software. The normality of data was analyzed with the Shapiro-Wilk test. Some data did not conform to a normal distribution, so the results were expressed as median [interquartile range (IQR)], and the Wilcoxon signed rank test was used to evaluate the statistical significance of the differences between groups. Spearman’s rank correlation test was conducted to examine correlations among variables. When the *p-*value was below 0.05, the result was considered statistically significant.

## 3 Results

### 3.1 Participants’ characteristics

The demographic and primary clinical characteristics of the participants are summarized in Table 1. All six participants were residents of Chengdu, Sichuan, China, consisting of 3 males and 3 females, with a mean age of 55.17 ± 15.6 years and a mean body mass index (BMI) of 23.20 ± 3.28 kg/m^2^. None of the participants had ever smoked or drunk alcohol. Regarding lifestyle habits, two participants (33.33%) drank water from reservoirs in plastic barrels, and two participants (33.33%) had the habit of consuming take-out food. Clinically, two out of the six participants (33.33%) reported symptoms of diarrhea, and one participant (16.67%) had comorbid underlying diseases. Supplementary Table 2 contains comprehensive questionnaire data; Supplementary Table 3 shows the participants’ basic information, lifestyle choices, and main clinical manifestations; and Supplementary Table 4 summarizes key clinical data about intestinal inflammation and erosion, such as hemoglobin levels, concentrations of high-sensitivity C-reactive protein (hs-CRP), and endoscopic findings.

**TABLE 1:** Demographic characteristics of the participants in this study (*n* = 6).

Characteristics	Categories	Values
Age (years), Mean ± SD		55.17 ± 15.6
Sex, *n* (%)	Male	3 (50.00)
	Female	3 (50.00)
BMI (kg/cm^2^), Mean ± SD		23.20 ± 3.28
Smoking status, *n* (%)	Yes	0(0)
	No	6(100.00)
Alcohol-drinking, *n* (%)	Yes	0(0)
	No	6(100.00)
Water source, *n* (%)	Reservoir with plastic barrel	2(33.33)
	Reservoir without plastic barrel	4(66.67)
Take-out consumption, *n* (%)	Yes	2(33.33)
	No	4(66.67)
Symptom, *n* (%)	Yes	
	Diarrhea	2(33.33)
	No	4(66.67)
Underlying disease, *n* (%)	Yes	1(16.67)
	No	5(83.33)

BMI, body mass index; *n* (%), numbers and percentages.

### 3.2 Pathological comparison between normal and erosive intestinal tissues

**FIGURE 1:**
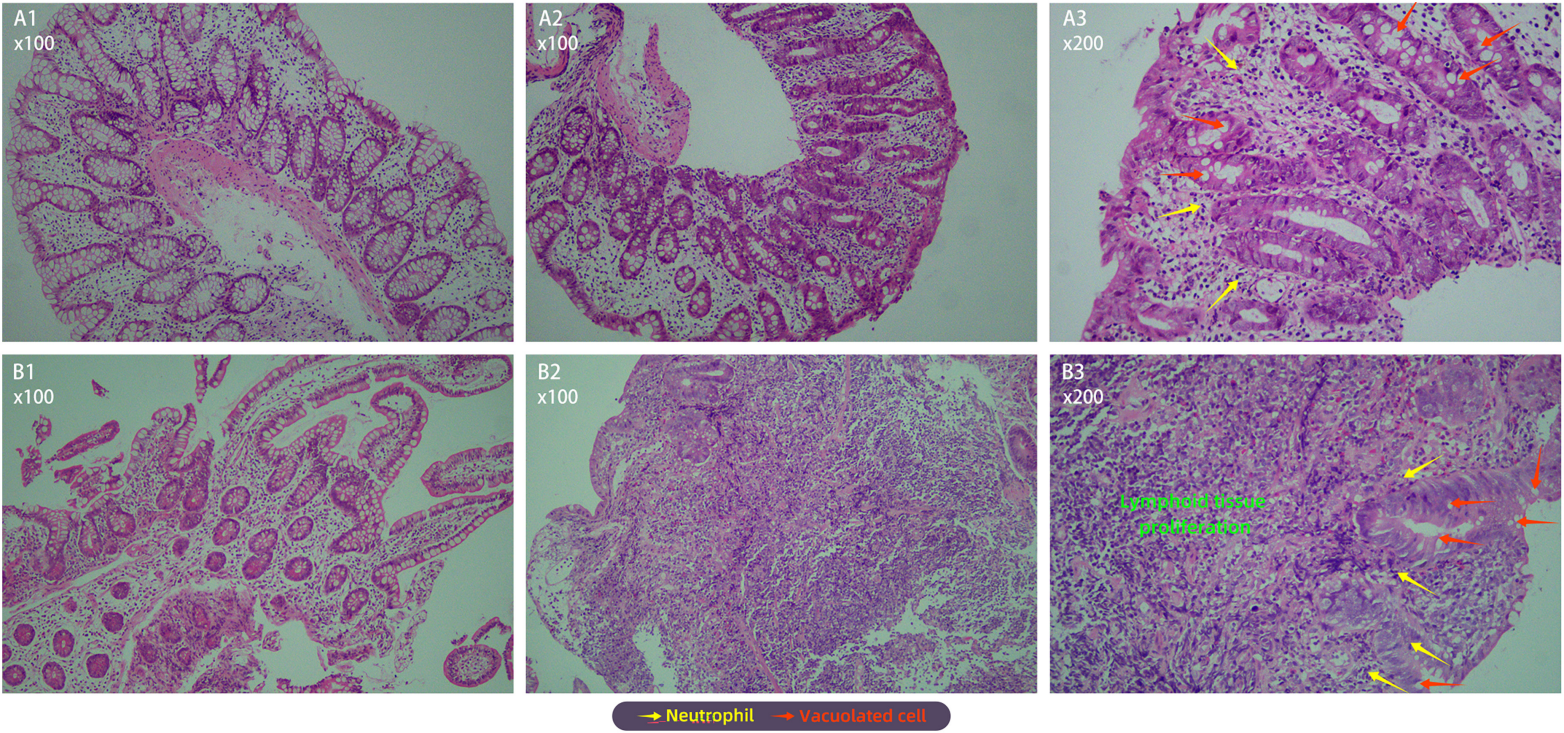
Representative H&E-stained pathological micrographs of the colon and terminal ileum. **(A)** Colon tissue from volunteer 1. **(B)** Terminal ileal tissue from volunteer 4. Panels 1 (all) show non-inflamed tissues at 100× magnification; Panels 2 (all) show inflamed tissues at 100× magnification; Panels 3 (all) show inflamed tissues at 200× magnification. Neutrophilic infiltration, appearance of vacuolated cells, and lymphoproliferation were observed in A3 and B3. The finger-like villi disappeared in B2.

Pathological examination of erosive tissue obtained by lower gastrointestinal endoscopy revealed mild to moderate inflammatory changes, including shortening and disappearance of intestinal villi, varying degrees of neutrophilic infiltration, cryptitis and pericryptitis, and reduction or even disappearance of intrinsic glands (Figure 1). Pathological images of the remaining volunteers are presented in Supplementary Figure 1.

### 3.3 Occurrence of MNPs in intestine

**FIGURE 2:**
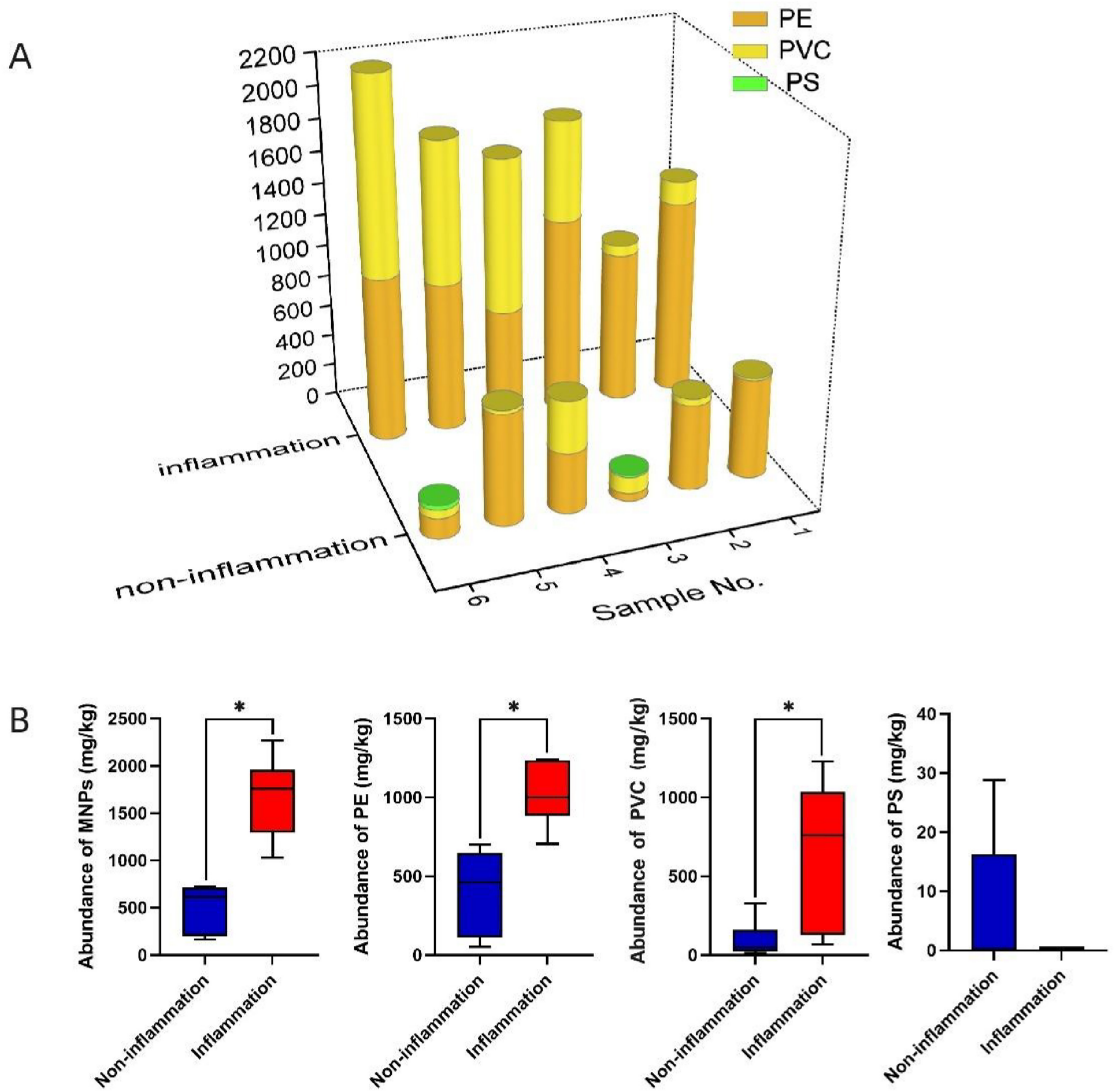
Characteristics of intestinal MNPs detected by Py-GC/MS. **(A)** Types and abundance of MNPs in each sample, with different colors representing different types of MNPs. **(B)** Comparison of total MNPs abundance, PE abundance, PVC abundance, PS abundance, and number of MNPs types (from left to right) between the non-inflammatory and inflammatory groups, with different colors representing different groups. The upper and lower horizontal lines of box plots represent the maximum and minimum values, the upper and lower edges represent the upper and lower quartiles, and the middle horizontal line represents the median. *p < 0.05.

For the first time, Py-GC/MS was used to measure MNPs in human intestinal tissues. The findings confirmed that polyethylene (PE) and polyvinylchloride (PVC) were widely present in all target tissues, and polystyrene (PS) was found in only two of the purposeful tissues under investigation (Figure 2A and Supplementary Table 4). There were no statistically significant differences in concentrations of PE, PVC, PS, or total MNP between colonic and ileal tissues within the same group (*p* > 0.05). The concentrations of PE, PVC, and total MNPs in inflammatory tissues were 1000.78 (351.23) mg/kg, 759.51 (862.97) mg/kg, and 1758.15 (664.07) mg/kg, respectively, which were significantly higher than those in non-inflammatory tissues [PE: 460.28 (535.46) mg/kg; PVC: 47.21 (138.16) mg/kg; total MNPs: 611.49 (510.64) mg/kg; *p* = 0.028] (Table 2). As illustrated in Figure 2B and Table 2, MNP concentrations in erosive inflammatory intestinal tissues were consistently higher than those in non-inflammatory tissues across all subjects. Additionally, the total ion chromatogram of all samples is presented in Supplementary Figure 2. Supplementary Figure 3 displays representative extracted ion chromatograms and mass spectra of multiple MNPs.

**TABLE 2:** Content of the MNPs extracted from the intestine tissues.

Groups	MNP abundance (mg in 1 kg intestinal tissue), Median (IQR)			
	PE	PVC	PS	Total
Non-inflammatory intestinal tissues	460.28 (535.461)	47.21 (138.16)	0 (22.26)	611.49 (510.64)
Inflammatory intestinal tissues	1000.78 (351.23)	759.51 (862.07)	0 (0)	1758.15 (664.07)
*p*-value	0.028	0.028	0.18	0.028

PVC, polyvinyl chloride; PE, polyethylene; PS, polystyrene. “Total” means the sum of PVC, PE, and PS.

### 3.4 Influence of living habits, clinical symptoms, and underlying diseases on MNP concentrations

**FIGURE 3:**
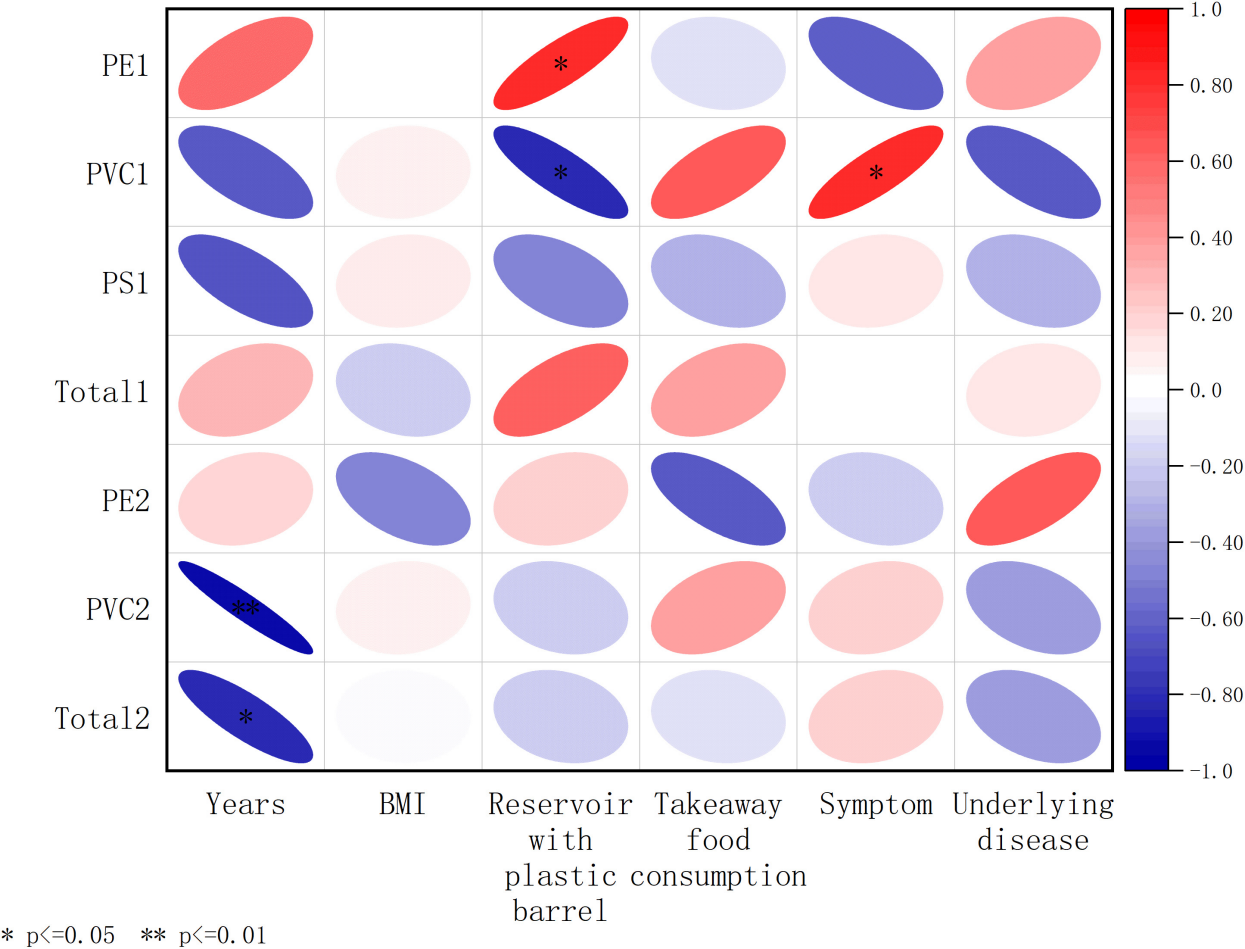
Heat map summarizing the correlation of micro- and nanoplastics and the years, BMI (body mass index), living habits and symptoms, and underlying disease. PVC, polyvinyl chloride; PE, polyethylene; Total, the total content of micro- and nanoplastics. Group 1 is non-inflammatory tissues, while group 2 is inflammatory tissues. *p < 0.05, **p < 0.01.

Spearman’s rank correlation analysis was used to investigate the associations between six variables (age, BMI, consumption of reservoir water stored in plastic barrels, takeaway food intake, clinical symptoms, and underlying diseases) and the concentrations of PE, PVC, PS, and total MNPs. Supplementary Table 5 provides correlation coefficients (ρ), exact *p*-values, and 95% CI for each correlation result. The results indicated that in non-inflammatory tissues, the presence of diarrheal symptoms might lead to an increase in PVC concentrations (ρ = 0.828, *p* = 0.042), while drinking water from a reservoir with a plastic barrel was associated with a reduction in PVC concentrations (ρ = −0.828, *p* = 0.042) and an amplification in PE concentrations (ρ = 0.828, *p* = 0.042). In inflammatory intestinal tissues, age had a negative correlation with PVC concentrations (ρ = −0.943, *p* = 0.005) and total MNP concentrations (ρ = −0.829, *p* = 0.042). MNP concentrations and comorbidities (including diabetes, renal insufficiency, and hypertension) did not significantly correlate (*p* > 0.05). Furthermore, no clear correlations between intestinal MNP concentrations, BMI, and takeaway food consumption were discovered (Figure 3).

## 4 Discussion

To the best of our knowledge, this is the first study to quantify MNPs in paired inflammatory and non-inflammatory human intestinal tissues using Py-GC/MS. Total MNP concentrations were 611.49 (510.64) mg/kg in non-inflammatory tissues and 1758.15 (664.07) mg/kg in inflammatory tissues, with significantly higher levels observed in inflamed mucosa. There were possible associations found between intestinal MNP accumulation and age, takeaway food consumption, bottled water intake, or diarrheal symptoms.

Notably, the following interpretations must be treated with considerable caution, as this investigation represents a preliminary exploratory investigation with a small sample size and no independent healthy control group. Integrating existing literature with our novel observational data, the subsequent discussion elaborates on plausible explanations for our findings, highlights consistencies and discrepancies with prior research, and systematically addresses the study’s limitations and directions for future research.

### 4.1 Types and concentrations of MNPs in gut tissues

Supplementary Table 6 provides a systematic summary of current studies on MNPs in the human gastrointestinal tract. Polycarbonate, polypropylene, and polyamide particles have been detected in human colon tissues (17). A comparison of MNP profiles in tumor and non-tumor colonic tissues revealed that the most common plastic particle types were PE, polymethyl methacrylate (PMMA), and nylon (PA) (4). However, these findings are inconsistent with the results of this study, which showed that PE and PVC were the predominant MNP types in colonic and terminal ileal tissues, while PS was detected in just two samples.

Polyethylene is extensively utilized in packaging materials, water bottle caps, and household supplies (18), whereas PVC is commonly used in textiles, footwear, interior furnishings, cable insulation compounds, and numerous other daily-use items (19). Frequent human exposure pathways, such as drinking reservoir water transported via plastic pipelines and takeaway food intake, may contribute to gastrointestinal MNP ingestion.

Except for types, particle mass was normalized to 0.8–1.4 ng per plastic particle for standardized cross-study comparisons of MNP concentrations (20). The MNP concentrations detected in this study were much higher than those reported in prior investigations. Variations in cohort selection, anatomical sampling sites, tissue processing protocols, and analytical methodologies could be the source of these disparities. Lifestyle choices and disease status may also have an impact on MNP’s content (13, 21).

First, in contrast to previous studies, participants with severe conditions such as tumors were excluded from the cohort. Tumor presence has been shown to change intestinal mucosal architecture and inflammatory profiles (22), which may have an impact on MNP translocation across the mucosal barrier (23).

Second, unlike the full-thickness colon tissue obtained by surgical excision in previous studies, the intestinal tissue samples in this research were collected from the colon and terminal ileum using biopsy forceps. Histological analyses confirmed that the intestinal tissues obtained by the biopsy forceps were primarily restricted to the mucosal layer and did not typically extend into the submucosa. The mucosal layer, muscularis mucosa, and submucosa—anatomical regions containing the intestinal mechanical barrier—have been shown in earlier animal studies to be the main locations of inflammatory damage and pathological changes brought on by MNPs (24–27).

Third, the specimen processing procedures used in this study differ from those used in earlier research. In this study, MNPs were extracted from intestinal tissues using an extraction method based on the solubility of different MNPs in different solvents; however, conventional treatment with strong acid and alkali may destroy some of the MNPs (28), resulting in an underestimation of actual MNP burdens in biological samples.

Lastly, there are differences in detection methods. Currently, the most widely utilized techniques for MNP detection are visual inspection, Fourier Transform Infrared (FTIR) spectroscopy, or Raman spectroscopy (29). However, these three techniques are susceptible to outside influences and are unable to distinguish between smaller MPs, particularly NPs (30, 31).

### 4.2 Association between MNPs and gut inflammation

Preclinical studies demonstrate that MNP exposure induces intestinal inflammation in aquatic organisms (32, 33) and elicits Crohn’s ileitis-like epithelial injury in mice (27). Clinical evidence similarly shows elevated fecal levels of MNPs and PVC in patients with inflammatory bowel disease (IBD) compared with healthy controls (5). Mechanistic studies in murine models indicate that MNPs can induce intestinal cell apoptosis, activate signaling pathways including NF-κB, promote pro-inflammatory cytokine release, disrupt intestinal tight junctions, increase mucosal permeability, and ultimately contribute to intestinal inflammatory injury (10).

Collectively, these studies indicate a potential connection between MNP exposure and intestinal inflammation. In this study, MNPs were directly detected in human intestinal mucosal specimens, and quantifiable MNP burdens in eroded intestinal tissues were found to be higher than those in histologically normal intestinal tissues. These preliminary observational data support a potential association between MNP accumulation and intestinal inflammation. However, due to the small sample size, no causal inferences can be drawn, and this association cannot be regarded as definitive. Further investigations with larger cohorts and in-depth mechanistic studies are therefore warranted to validate these findings.

### 4.3 Association between MNPs and disease

In the present study, MNP concentrations in both non-inflammatory and inflammatory intestinal tissues were associated with diarrheal symptoms, whereas no significant correlations were observed between MNP levels and underlying comorbidities. These observations indicate that specific MNP subtypes might be connected to gastrointestinal symptoms. Numerous pathophysiological mechanisms, primarily categorized as secretory, osmotic, inflammatory, intestinal transit dysfunction, and reduced functional absorptive capacity, are responsible for diarrhea (34). MNP exposure impairs intestinal mucus secretion, damages the mucosal barrier, increases small intestinal permeability, and disrupts the expression of ion transporters, all of which are pathophysiological processes that collectively compromise intestinal water and electrolyte reabsorption, according to earlier experimental studies conducted in murine models. Notably, it has been demonstrated that these disruptions cause mice to consume less food and become constipated (10); in other studies, mice exposed to MNP also showed persistent constipation (35). Although there hasn’t been much research done on the connection between MNPs and human disease, what is known suggests that MNPs may be associated with intestinal disorders such as colorectal tumors (21) and IBD (5). These investigations support our findings, indicating that PVC may be involved in the association between MNP exposure, intestinal inflammation, and diarrheal symptoms. Nevertheless, these observations remain preliminary and require validation in larger, adequately powered cohort studies.

Previous studies have indicated that MNPs may be associated with glucose and lipid metabolism (36), hypertension (37), and even an elevated risk of major adverse cardiovascular events in patients with acute myocardial infarction (38). On the other hand, our study failed to find any significant connections between intestinal MNP burdens and systemic comorbidities, specifically diabetes mellitus, renal insufficiency, and hypertension. With only one member of our cohort having pre-existing systemic comorbidities, this discrepancy is likely due to the study’s small sample size. To validate these findings and elucidate the potential mediating role of the gut microbiota in the cascade of systemic pathological effects linked to intestinal MNP exposure, future well-powered prospective cohort studies are crucial.

### 4.4 The influence of living habits and years on MNP content

In this study, elevated PE concentrations and reduced PVC concentrations were detected in non-inflammatory intestinal tissues of adults consuming reservoir water stored in plastic barrels or bottled water. PE is a primary component of bottled water packaging materials (18),whereas PVC is widely used in water supply pipelines (39). These results suggest that different drinking water sources may contribute to distinct patterns of MNP exposure. A person’s lifestyle choices may influence their exposure to MNPs (40), as prior research has shown that MNPs can be found in food, drinks, cosmetics, air, and salt (41). Variations in MNP types and concentrations may arise from different drinking practices (40). Although our results did not show an association between takeaway food consumption and intestinal MNP burdens, a previous study has found that daily use of plastic products may affect the release of plastic particles (42). MNPs in plastic take-out containers are caused by plastic abrasion and air deposition on the container surface; MNP intake increases with takeout food consumption. (42) The amount of microplastics in human feces and the composition of fecal flora in human feces have also been found to change when specific food sources are consumed. (13) The inconsistent results between our study and previous reports may be due to the limited sample size, as well as the difference between measuring external MNP intake and internal MNP levels in intestinal tissues.

Adults have a significantly higher MNP intake compared to children; some of the plastics are eliminated through biliary or fecal excretion, while the remaining amount builds up throughout the body (43, 44). Long-term accumulation has been associated with cellular and mucosal damage *in vitro* (45) and *in vivo* (46). Contrary to earlier research, the results further suggest that PVC and total MNP concentrations in inflammatory intestinal tissues decrease with age. This discrepancy is likely due to the small sample size and the focal inflammatory state of the sampled tissues, which may modify age-related accumulation patterns.

### 4.5 Limitations of the study

Although the validity of our results has been verified through a comprehensive QA/QC framework, this study still presents several notable limitations that should be clearly acknowledged. First, the present study included only six participants, which resulted in limited statistical power and constrained our ability to draw definitive conclusions. The small sample size may also limit the generalizability of our findings, as these results may not fully represent the broader population. Accordingly, our findings should be interpreted as preliminary and exploratory in nature, rather than conclusive evidence. Future studies employing larger and more diverse cohorts will be necessary to validate these observations, improve statistical reliability, and strengthen the generalizability of the results. Second, this study lacks a genuinely healthy control group. In order to minimize inter-individual variability, paired comparisons were given priority for patients with intestinal erosion; however, any comparison of MNP profiles between patients with intestinal erosion and healthy subjects was not possible due to the absence of healthy control groups. As a result, it is more difficult to determine whether MNPs and the onset of intestinal inflammation are causally or correlatively related. Despite these limitations, the preliminary dataset from this exploratory study offers useful recommendations to guide the planning and execution of large-scale trials in later studies in this area.

## 5 Challenges and perspectives

Micro- and nanoplastic exposure in the human living environment has greatly increased due to the widespread production and use of plastics, posing a risk to human health (18). Most commonly, contaminated food is how MNPs enter the human body and reach the intestinal tract. Smaller MNPs may cross the intestinal barrier, enter the bloodstream, and subsequently disseminate to other tissues and organs (8, 47). Previous studies using fish and murine models have shown that MNPs that are not cleared in a timely manner often build up in the intestinal tract, causing inflammation and mucosal damage (48). Although this study provides preliminary evidence supporting an association between MNPs and intestinal inflammation, it has several limitations: the sample size is relatively small; detailed characterization of MNPs (including particle shape, size, and other morphological properties) is lacking; the molecular mechanisms underlying MNP-induced toxicity were not further explored; and only the influence of short-term lifestyle habits on intestinal MNP levels was considered. Therefore, the potential long-term health risks of lifestyle habits on intestinal function warrant further investigation.

To clarify the possible negative effects of MNP exposure on the human digestive system and the underlying pathogenic mechanisms connecting MNPs to related diseases, more research is necessary. In order to thoroughly investigate the molecular mechanism by which MNPs cause intestinal inflammation, we plan to expand the sample size and combine animal models and cell experiments to deeply explore the molecular mechanism in the future. In addition, epidemiological studies should be carried out to evaluate the association between MNP exposure and intestinal disorders such as IBD and irritable bowel syndrome so as to provide a scientific basis for the formulation of relevant public health policies. It is crucial to create innovative formulations that can mitigate MNP accumulation in the human body, remove pre-existing MNP deposits, prevent and treat related diseases, and ultimately protect human health in addition to lowering MNP ingestion through optimized lifestyle practices and reduced usage of high-risk plastic products.

## 6 Conclusion

To the best of our knowledge, Py-GC/MS was utilized for the first time to demonstrate the presence of MNPs in human intestinal tissues. The median MNP level in inflammatory intestinal tissues was statistically significantly higher than that in non-inflammatory intestinal tissues (*p* < 0.05), with respective concentrations of 1758.15 (664.07) mg/kg and 611.49 (510.64) mg/kg. The two main MNP types identified were PVC and PE, whereas PS was detected in only two samples. This study provides preliminary evidence supporting a potential association between MNP accumulation and intestinal inflammation, based on the observation of differences in MNP levels between inflammatory and non-inflammatory intestinal tissues. Intestinal MNP levels may be associated with gastrointestinal symptoms, age, and lifestyle. Owing to the limited sample size, we could not reliably clarify the specific associations of underlying health status and lifestyle with intestinal MNP abundance, nor explore their potential pathogenic relevance. As an exploratory preliminary study, our findings highlight the need for larger-scale cohort studies to generate more representative data. Such investigations are warranted to validate the observed associations and enable a more comprehensive assessment of MNP exposure in the intestinal inflammatory tissues of patients with enteritis.

## Data Availability

The original contributions presented in this study are included in the article/Supplementary material, further inquiries can be directed to the corresponding author.
