# Supplementary material for: Higher abundance of micro- and nanoplastics in inflammatory tissues from patients with intestinal erosion
Source: Front Med (Lausanne). 2026 Jul 10;13:1732146. doi: 10.3389/fmed.2026.1732146 (PMC13398087; doi:10.3389/fmed.2026.1732146)
Supplement: Supplementary file 1 [file Table_1.docx]

**Higher abundance of** **micro- and nanoplastics in inflammatory tissues from patients with intestinal erosion**

Lixia Du ^1,*^, Xuan Song ^2^, Tianchao Deng ^3^, Xiaoqian Feng ^4^, Lijuan Qiao ^1^, Hui Xu ^1^, Lianlian Liu ^5^, Yan Xu ^5^, Ying Zeng ^5^, Hua Qing ^5^, Qin Li ^5^

^1^ Department of Gastroenterology, Chengdu BOE Hospital, Chengdu 610219, China

^2^ Center of Reproductive Medicine, Chengdu BOE Hospital, Chengdu 610219, China

^3^ Department of Pathology, Chengdu BOE Hospital, Chengdu 610219, China

^4^ Department of Comprehensive Pediatric Internal Medicine, Chongqing University Three Gorges Hospital, Chongqing 404010, China

^5^ Center of Endoscopy, Chengdu BOE Hospital, Chengdu 610219, China

***Corresponding author:**

Lixia Du, Department of Gastroenterology, Chengdu BOE Hospital, No. 1, Jingxing Road, Tianfu International Bio City, Chengdu, Sichuan,610219, China, E-mail: dulixia68@outlook.com

**Table S1** Py-GC/MS calibration curves of polymers…………………....…. S3

**Table S2** Questionnaire….………………………...............................…...… S4

**Table S3** Details of the study volunteers….…….…………………….......... S5

**Table S4** Detailed Py-GC/MS results for each sample…………….............. S6

**Table S5** The influence of lifestyle habits, clinical symptoms, and diseases on MNP contents….……………………………………………........................... S7—S8

**Table S6** MNPs found in human gut tissues.........................………………... S9

**Fig. S1** Pathological examination of the other four participants….….......... S10

**Fig. S2** Total ion chromatogram for each sample............................... S11—S12

**Fig. S3** Representative extracted ion chromatograms and mass spectrograms of

MNPs.................................................................................................... S13—S14

**Abbreviations and References**..................................................................... S15

| **Table S1 Py-GC/MS calibration curves of polymers** | | | | | | |
| --- | --- | --- | --- | --- | --- | --- |
| MNPs | Quantifier ion (m/z) | Quantitative curve equation | R^2^ | Characteristic fragments | LOQ(μg) | LOD |
| PVC | 128 | y = 4.2637x - 0.286 | 0.9970 | Naphthalene monomer | 0.06 | 0.020 |
| PS | 91 | y = 300.89x - 9.1689 | 0.9927 | styrene monomer | 0.02 | 0.007 |
| PE | 111 | y = 0.7378x - 14.383 | 0.9968 | Tridecene | 0.22 | 0.073 |

Abbreviation: LOQ, limits of quantification; LOD, limits of detection.

**Table S2.** **Questionnaire.**

| Questions | Options |
| --- | --- |
| Do you have any underlying medical conditions? If so, what is it? | Yes: __________（Details） |
|  | No: |
| Do you have any clinical symptoms? If so, what is it? | Yes: __________（Details） |
|  | No: |
| What is your water source, with or without plastic barrel? | _________________________ |
| Do you drink alcohol every week? If so, how much alcohol per week? | Yes:  __________mL |
|  | No |
| How many times do you eat takeaway food per week? | _____times |
| Do you smoke (including secondhand smoke)? If so, how many times per week? | Yes |
|  | No |
| Do you live or work near a plastic factory? | Yes |
|  | No |

**Table S3. Details of the study volunteers**

| Sample number | Gender | Age (years) | Height (cm) | Weight (kg) | BMI | Smoking | Alcohol-drinking | Water source | Takeaway food consumption | Symptom | Underlying disease |  |
| --- | --- | --- | --- | --- | --- | --- | --- | --- | --- | --- | --- | --- |
| 1 | Female | 68 | 156 | 45 | 18.49 | No | No | Reservoir with plastic barrel | No | Yes | Yes |  |
| 2 | Female | 72 | 151 | 64 | 28.07 | No | No | Reservoir without plastic barrel | No | Yes | No |  |
| 3 | Female | 52 | 155 | 50 | 20.81 | No | No | Reservoir without plastic barrel | No | No | No |  |
| 4 | Male | 38 | 175 | 72 | 23.51 | No | No | Reservoir without plastic barrel | Yes | No | No |  |
| 5 | Male | 65 | 178 | 76 | 23.99 | No | No | reservoir with plastic barrel | No | No | No |  |
| 6 | Male | 36 | 172 | 72 | 24.34 | No | No | Reservoir without plastic barrel | No | No | No |  |

- Smoking, alcohol-drinking, takeaway food, symptom: the frequencies of more than three days per week in the last year were defined as yes.

**Table S4 Detailed Py-GC/MS results for each sample (mg in per 1 kg intestinal tissue)**

| S.N. | Tissue |  | | Non-inflammatory intestinal tissues | | | Inflammatory intestinal tissues | | |
| --- | --- | --- | --- | --- | --- | --- | --- | --- | --- |
|  |  | PE | PVC | | PS | Total | PE | PVC | Total |
| 1 | Colon | 628.7166 | 13.5307 | | - | 642.2473 | 1238.9506 | 145.7074 | 1384.658 |
| 2 | Colon | 539.1056 | 41.6243 | | - | 580.7299 | 960.7688 | 68.0716 | 1028.8404 |
| 3 | Colon | 51.72 | 104.17 | | 12.08 | 212.28 | 1233.4834 | 623.4518 | 1856.9352 |
| 4 | Ileum | 381.4451 | 326.4256 | | - | 707.8707 | 705.6944 | 972.0934 | 1677.7878 |
| 5 | Ileum | 699.4929 | 24.2597 | | - | 723.7526 | 942.934 | 895.5751 | 1838.5091 |
| 6 | Ileum | 130.69 | 52.79 | | 28.8 | 167.97 | 1040.7864 | 1227.4957 | 2268.2821 |

| **Table S5** **The** **influence of lifestyle habits, clinical symptoms, and diseases on MNP contents** | | | | | | | | |
| --- | --- | --- | --- | --- | --- | --- | --- | --- |
|  |  |  | Years | BMI | Reservoir with plastic barrel | Takeaway food consumption | Symptom | Underlying disease |
| Non-inflammation tissues | PE | Correlation efficient | 0.6 | 0.029 | .828* | -0.131 | -0.621 | 0.393 |
|  |  | Significance (two-tailed) | 0.208 | 0.957 | 0.042 | 0.805 | 0.188 | 0.441 |
|  |  | N | 6 | 6 | 6 | 6 | 6 | 6 |
|  | PVC | Correlation efficient | -0.657 | 0.086 | -.828* | 0.655 | .828* | -0.655 |
|  |  | Significance (two-tailed) | 0.156 | 0.872 | 0.042 | 0.158 | 0.042 | 0.158 |
|  |  | N | 6 | 6 | 6 | 6 | 6 | 6 |
|  | PS | Correlation efficient | -0.676 | 0.101 | -0.49 | -0.31 | 0.122 | -0.31 |
|  |  | Significance (two-tailed) | 0.14 | 0.848 | 0.324 | 0.55 | 0.817 | 0.55 |
|  |  | N | 6 | 6 | 6 | 6 | 6 | 6 |
|  | Total | Correlation efficient | 0.314 | -0.2 | 0.621 | 0.393 | 0 | 0.131 |
|  |  | Significance (two-tailed) | 0.544 | 0.704 | 0.188 | 0.441 | 1 | 0.805 |
|  |  | N | 6 | 6 | 6 | 6 | 6 | 6 |
| Inflammation tissues | PE | Correlation efficient | 0.2 | -0.486 | 0.207 | -0.655 | -0.207 | 0.655 |
|  |  | Significance (two-tailed) | 0.704 | 0.329 | 0.694 | 0.158 | 0.694 | 0.158 |
|  |  | N | 6 | 6 | 6 | 6 | 6 | 6 |
|  | PVC | Correlation efficient | -.943** | 0.086 | -0.207 | 0.393 | 0.207 | -0.393 |
|  |  | Significance (two-tailed) | 0.005 | 0.872 | 0.694 | 0.441 | 0.694 | 0.441 |
|  |  | N | 6 | 6 | 6 | 6 | 6 | 6 |
|  | Total | Correlation efficient | -.829* | -0.029 | -0.207 | -0.131 | 0.207 | -0.393 |
|  |  | Significance (two-tailed) | 0.042 | 0.957 | 0.694 | 0.805 | 0.694 | 0.441 |
|  |  | N | 6 | 6 | 6 | 6 | 6 | 6 |
| Notes: “Total” means the sum of PVC, PE and PS. N: sample size; **p＜*0.05*,* ***p＜*0.01. | | | | | | | | |

| **Table S6** **MNPs found in human gut tissues**   \| Specimen \| Detection method \| MNPs \| \| \| \| References \| \| --- \| --- \| --- \| --- \| --- \| --- \| --- \| \| Types \| Shape \| Size \| Contents \| \| colorectal cancer and non-cancer \| Stereo microscope, FTIR \| PC, PP, PA \| Filaments or fibers \| 0.8 to 1.6 mm \| 28.1±15.4 particles/g \| [1] \| \| Tumor and non-tumor colon \| Light microscope and ATR-FTIR, Raman \| PE, PMMA, Nylon (PA) \| - \| 1 to 1299 μm \| 207.78 ± 154.12 to 702.68 ± 504.26 particles/g \| [2] \| |
| --- | --- | --- | --- | --- | --- | --- | --- | --- | --- | --- | --- | --- | --- | --- | --- | --- | --- | --- | --- | --- | --- | --- | --- | --- | --- |

**Fig. S1**


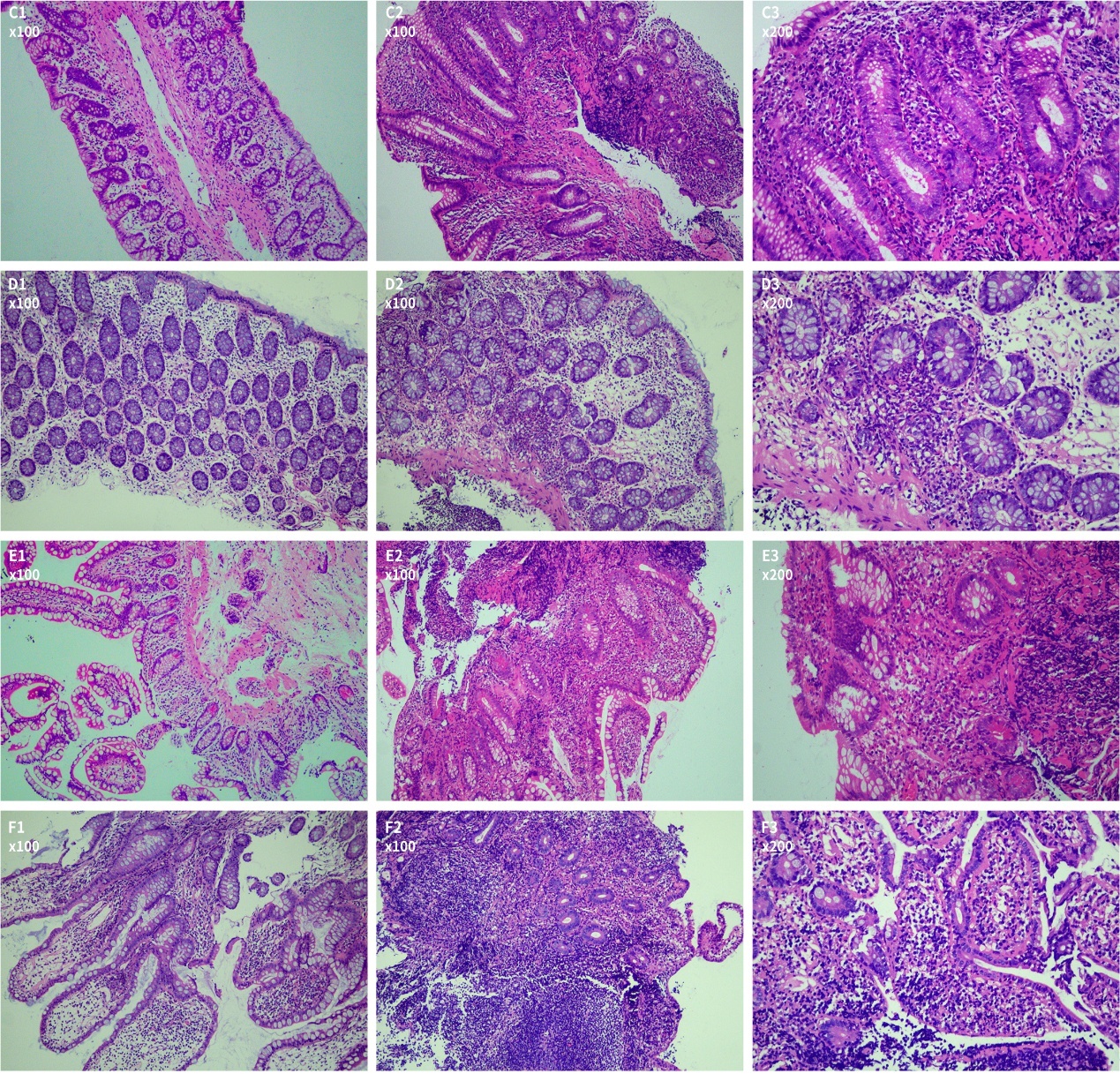
**Fig. S1.** Pathological examination of the other four participants. C-D are from colonic tissues. E-F are from terminal ileum. All Figures 1 are non-inflammatory tissues observed at 100× magnification, all Figures 2 are inflammatory tissues observed at 100× magnification, all Figures 3 are inflammatory tissues observed at 200× magnification.

**Fig. S2**


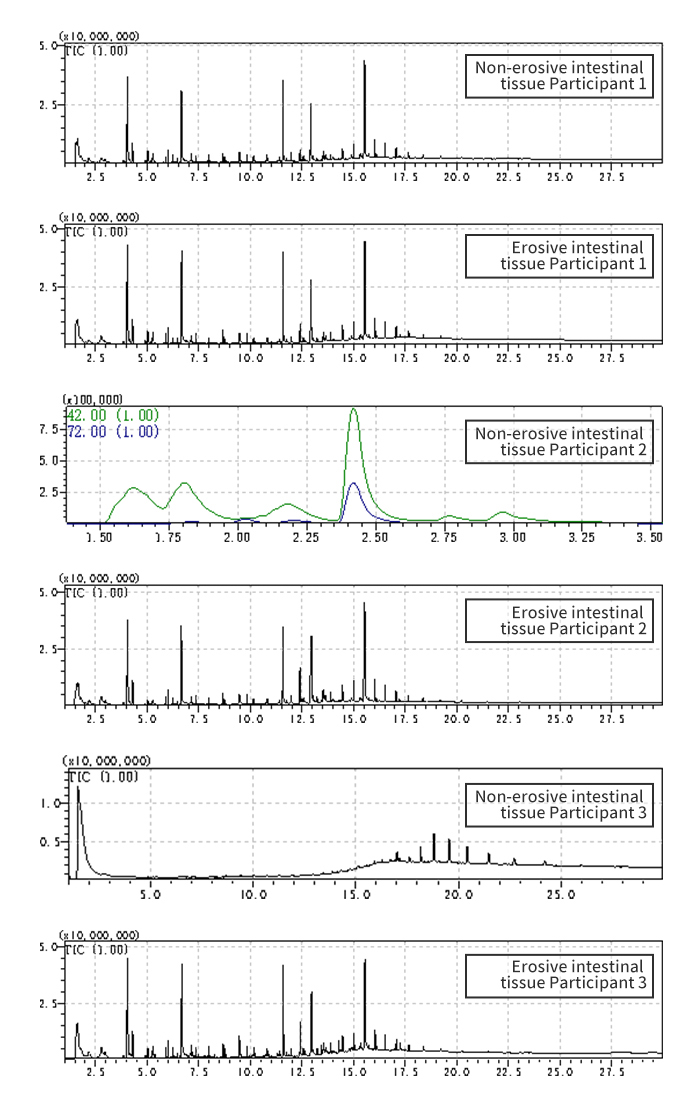


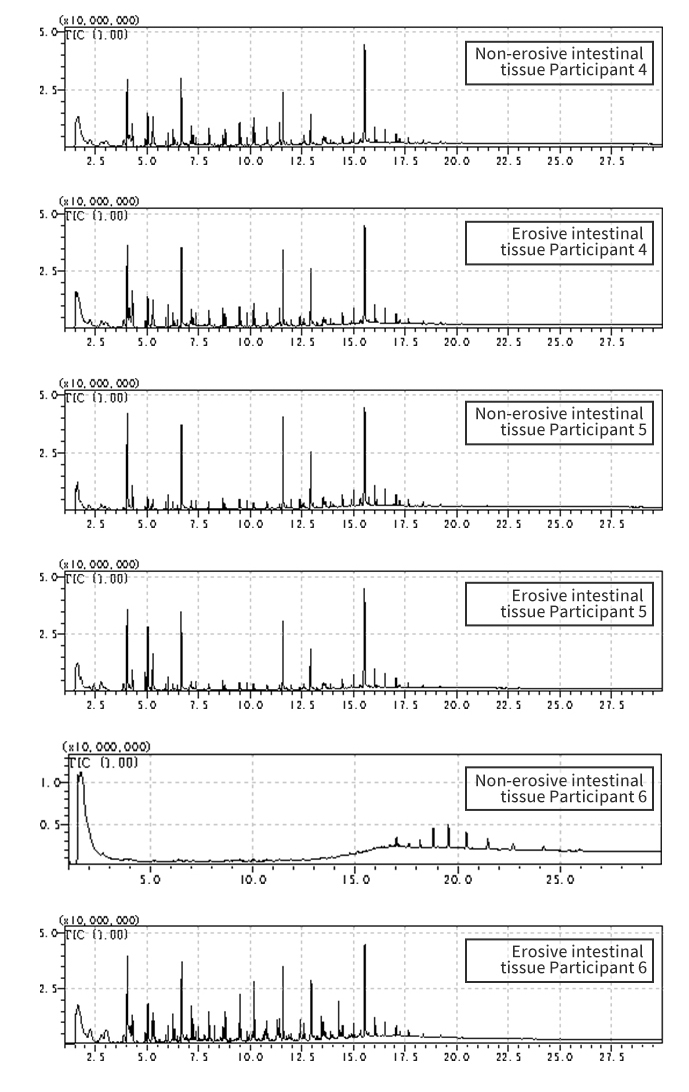


**Fig. S2.** The total ion chromatogram for each sample.


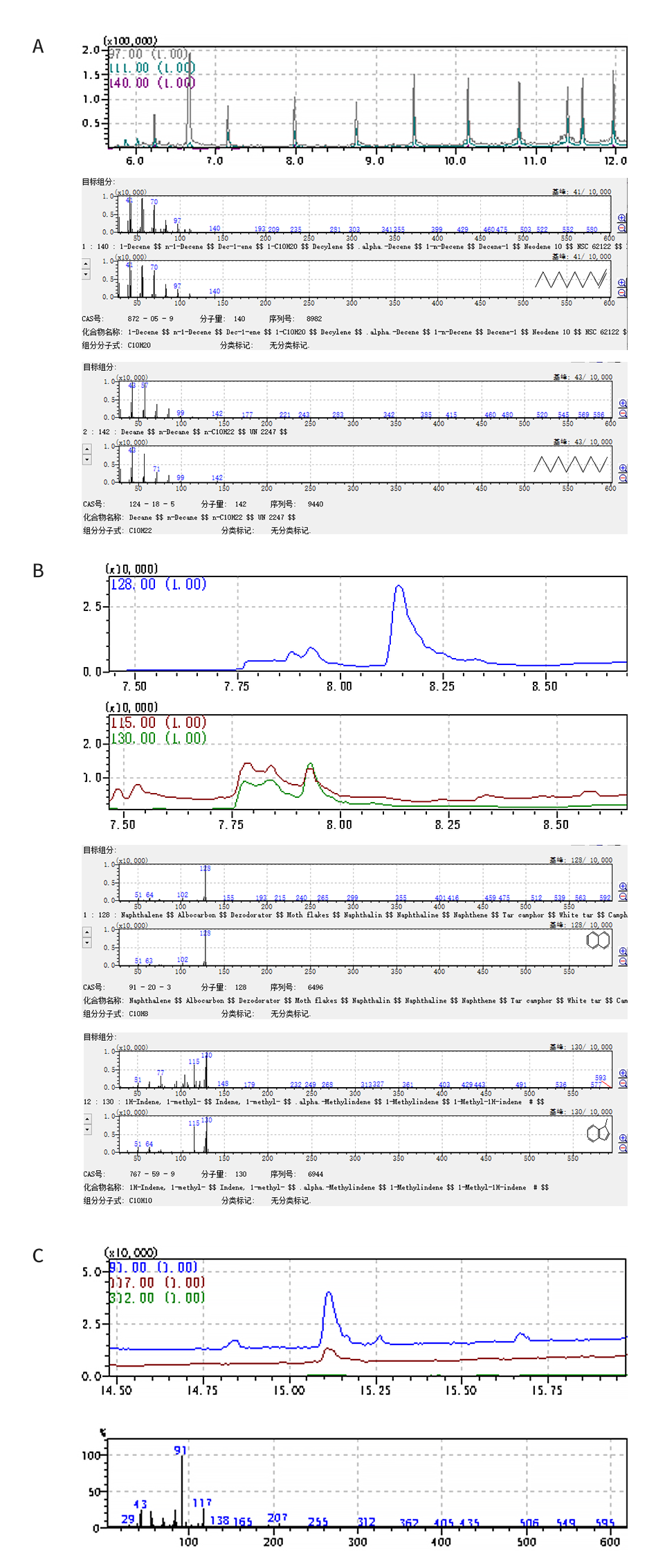


**Fig. S3.** **Representative** **extracted ion chromatograms and mass spectrograms of MNPs.** (A–C) The extracted ion chromatograms are on the top and the mass spectrograms are on the bottom. (A) PE from inflammatory intestinal tissue of Participant 1, retention time: 6.231 min. (B) PVC from inflammatory intestinal tissue of Participant 5, retention time: 8.131 min. (C) PS from non-inflammatory intestinal tissue of Participant 6, retention time: 15.11 min.

**Abbreviations:** FTIR, Fourier Transform Infrared; ATR-，attenuated total reflection-; Py/GC-MS, pyrolysis gas chromatography/mass spectrometry; PP, Polypropylene; PE, Polyethylene; PVC, Polyvinyl chloride; PS, Polystyrene; PA, Polyamide; PC, polycarbonate.

**References:**

1. Ibrahim YS, Tuan Anuar S, Azmi AA, Wan Mohd Khalik WMA, Lehata S, Hamzah SR, et al. Detection of microplastics in human colectomy specimens. JGH Open [Internet]. 2020 [cited 2024 Jan 16];5:116–21. Available from: https://www.ncbi.nlm.nih.gov/pmc/articles/PMC7812470/

2. Cetin M, Demirkaya Miloglu F, Kilic Baygutalp N, Ceylan O, Yildirim S, Eser G, et al. Higher number of microplastics in tumoral colon tissues from patients with colorectal adenocarcinoma. Environ Chem Lett [Internet]. 2023 [cited 2024 May 11];21:639–46. Available from: https://link.springer.com/10.1007/s10311-022-01560-4
